# Introducing Thermal Wave Transport Analysis (TWTA): A Thermal Technique for Dopamine Detection by Screen-Printed Electrodes Functionalized with Molecularly Imprinted Polymer (MIP) Particles

**DOI:** 10.3390/molecules21050552

**Published:** 2016-04-26

**Authors:** Marloes M. Peeters, Bart van Grinsven, Christopher W. Foster, Thomas J. Cleij, Craig E. Banks

**Affiliations:** 1 Faculty of Science and Engineering, School of Science and the Environment, Division of Chemistry and Environmental Science, Manchester Metropolitan University, Chester Street, Manchester M1 5GD, UK; cwfoster90@gmail.com (C.W.F.); c.banks@mmu.ac.uk (C.E.B.); 2 Maastricht Science Programme, Maastricht University, P.O. Box 616, 6200 MD Maastricht, The Netherlands; bart.vangrinsven@maastrichtuniversity.nl (B.v.G.); thomas.cleij@maastrichtuniversity.nl (T.J.C.)

**Keywords:** molecularly imprinted polymers (MIPs), biomimetic sensors, heat-transfer method (HTM), thermal wave transport analysis (TWTA), neurotransmitters, screen-printing technology, cyclic voltammetry

## Abstract

A novel procedure is developed for producing bulk modified Molecularly Imprinted Polymer (MIP) screen-printed electrodes (SPEs), which involves the direct mixing of the polymer particles within the screen-printed ink. This allowed reduction of the sample preparation time from 45 min to 1 min, and resulted in higher reproducibility of the electrodes. The samples are measured with a novel detection method, namely, thermal wave transport analysis (TWTA), relying on the analysis of thermal waves through a functional interface. As a first proof-of-principle, MIPs for dopamine are developed and successfully incorporated within a bulk modified MIP SPE. The detection limits of dopamine within buffer solutions for the MIP SPEs are determined via three independent techniques. With cyclic voltammetry this was determined to be 4.7 × 10^−6^ M, whereas by using the heat-transfer method (HTM) 0.35 × 10^−6^ M was obtained, and with the novel TWTA concept 0.26 × 10^−6^ M is possible. This TWTA technique is measured simultaneously with HTM and has the benefits of reducing measurement time to less than 5 min and increasing effect size by nearly a factor of two. The two thermal methods are able to enhance dopamine detection by one order of magnitude compared to the electrochemical method. In previous research, it was not possible to measure neurotransmitters in complex samples with HTM, but with the improved signal-to-noise of TWTA for the first time, spiked dopamine concentrations were determined in a relevant food sample. In summary, novel concepts are presented for both the sensor functionalization side by employing screen-printing technology, and on the sensing side, the novel TWTA thermal technique is reported. The developed bio-sensing platform is cost-effective and suitable for mass-production due to the nature of screen-printing technology, which makes it very interesting for neurotransmitter detection in clinical diagnostic applications.

## 1. Introduction

Molecularly Imprinted Polymers (MIPs) are synthetic receptors that contain nanocavities with high affinity for their respective template molecules [1,2]. These ‘plastic’ antibodies are able to measure targets varying from small ions to larger cells within complex matrices. Over recent years, there has been a growing interest in the utilization of MIPs for diagnostic medical applications due to their straightforward production process and excellent stability under extremes of pH and temperature [3,4]. However, implementation of these particles into a sensing platform is a complicated task and traditional transduction techniques require bulky and expensive equipment, which does not make them suitable for incorporation into a portable set-up [5,6]. Examples of dopamine MIP chemosensors in the literature include their combination with gravimetric techniques, such as the quartz crystal microbalance [7], or sensors based on surface acoustic waves [8] and surface plasma resonance [9]. Peeters *et al.* [10] reported the detection of neurotransmitters with MIPs by using the heat-transfer method (HTM). Van Grinsven *et al.* [11], who discovered that melting of DNA induced a significant increase in the thermal resistance at the interface, first introduced this technique in 2012. For an overview of all recent applications, including the screening of cancer cells and detection of proteins, readers may consult references [10,11,12].

To measure MIP samples with HTM, first an adhesive polymer layer is coated on electrodes onto which MIP powders are applied with a stamp [13,14]. Through gentle heating of the substrate, the particles partially sink into the layer where they remain firmly fixated [15]. The main drawbacks of the described functionalization procedure are lack of control over particle distribution and the long preparation time. An interesting alternative would be to combine the functionalization of MIPs with screen-printing technology; a well-established technique for the fabrication of biosensors with a number of commercial applications, such as the glucose biosensor [16,17]. Screen-printed electrodes are cost-effective, the obtained electrode structures are highly reproducible, and the technique can be used for mass-production within portable devices [18,19]. There are a few examples of incorporating MIP particles *onto* screen-printed electrodes (SPEs) reported in the literature. A similar procedure as described before, applying MIP particles onto a polymer adhesive layer, was followed by Croux *et al.* [20] Other functionalization methods include immersing SPEs into a suspension with MIP microparticles [21] and coating by electro-polymerization [22,23]. A first attempt to mix the MIP particles with the ink was done by Kirsch *et al.* [24] who fabricated a sensor for the detection of polyhydrocarbons. A commercially available “D14” ink was added to a glass vial containing MIP particles and cyclohexanone and after thorough mixing, the solvent was evaporated in a vacuum oven at 60 °C overnight. Subsequently, this was spread over an area of the SPE defined with a mask of 3 mm diameter and dried for 1 h to modify the bulk of the electrodes with MIPs. All the described procedures require multiple preparation steps and are time-consuming. In addition, for all described bio-sensing platforms, detection limits were in the micromolar regime, which is often too high for relevant biological samples.

In this article we present a novel concept for the functionalization of biomimetic layers to produce bulk modified SPEs achieved by screen-printing a standard three electrode configuration and then printing an additional carbon layer that has been modified by directly mixing MIP particles into the bulk of the screen-printing ink. As a first proof-of-application, MIPs for dopamine are developed similar to literature procedures [25,26] and optimized by optical batch rebinding experiments. After determining its specificity and selectivity towards the template, the particles are functionalized within the SPEs by mixing them into different ratios with the screen-printing ink. Dopamine is selected because this neurotransmitter is indispensable for the efficient functioning of a variety of physiological actions [27]. Normal concentrations vary from the nanomolar range of serum to approximately 0.5–10 μM in other bodily fluids such as urine [28]. Abnormalities in dopamine levels are associated with psychiatric disorders such as schizophrenia and depression [27,29]. The dopamine content of certain foods, such as cheese and banana, is even considerably higher (mg dopamine/kg sample), and therefore it is interesting to study concentrations within food-related samples [30]. Electrochemical methods are commonly used as transduction techniques for this neurotransmitter due to its distinct oxidation signal [31]. First, dopamine concentrations within buffer solutions are measured with the bare SPEs and MIP-SPE via cyclic voltammetry. In the case for both electrodes, the limits of detection are found to be around 4 × 10^−6^ M, which is not sufficient to analyze all the relevant biological samples, and therefore thermal techniques will be employed to improve the performance of the sensor.

To our knowledge, this is the first time that thermal detection of MIP-SPE is reported. At first, HTM is used which relies on keeping the heat sink at a fixed temperature and measuring the temperature within the liquid, which is affected by the binding of dopamine to the MIP particles. Second, a novel thermal detection method is presented based on thermal wave transport analysis (TWTA). This technique sends a thermal wave through the functional interface and binding will affect both the amplitude and phase shift of the measured output signal in the liquid. Advantages over HTM include that it provides spatial information about the target-receptor dynamics and that it has the potential to improve signal-to-noise ratio since no stringent control of the heat sink temperature is required. In addition, its proof-of-application is demonstrated by measuring a relevant food sample.

In summary, a significant improvement is made with the novel functionalization strategy that involves direct mixing of the MIP particles within the bulk screen-printing ink. Thermal detection of MIP-SPE demonstrated enhancement of the detection limit with one order of magnitude compared to cyclic voltammetry. A novel technique is presented based on thermal waves, and first measurements indicated its potential for measuring not only buffer solutions, but also samples that are more complex. Through improving both the functionalization procedure and the sensing platform, in combination with the generic nature of the described biosensing platform, this is a first step towards a MIP-based chemosensor suitable for the commercial detection of neurotransmitters within complex matrices.

## 2. Results and Discussion

### 2.1. Batch Rebinding Experiments

**Figure 1:**
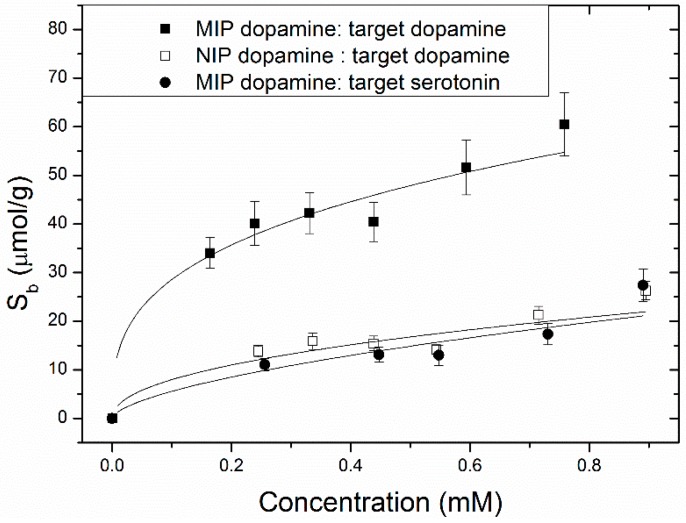
Binding isotherms of MIP (solid squares) and the corresponding NIP (open squares) upon exposure to aqueous dopamine solutions. To test the selectivity, the response of the MIP to serotonin was evaluated (filled circles). Solid lines are based on the allometric fit described in the text (*R*^2^ = 0.95); the imprinting factor for optimized monomer blend is close to 3. Error bars were calculated over the results of three individual experiments.

For the optical batch rebinding experiments, 20 mg of MIP- or NIP powder were added to 5 mL of aqueous dopamine or serotonin solutions with concentrations between 0.3 and 1 mM. After the suspensions were shaken for 12 h, the solutions were filtered and binding isotherms were constructed by plotting the free concentration *C_f_ vs.* the amount of template bound per g of the MIP or NIP, *S_b_* (Figure 1). All experiments were performed in triplicate.

From Figure 1, it is directly clear that there is a significant difference in binding between the MIP and its reference, the NIP. To determine the specificity, the imprint factor (IF) was used which is the amount bound to the MIP divided over that of that NIP at one specific concentration. The binding isotherms were fitted with a two-parameter fit of the following type to analyze the imprint factor at a specific concentration (Equation (1)):

(1) $$S_{b}\;=\;A\;\cdot \;C_{f}^{\nu }$$

where *S_b_* (μmol/g) stands for the amount of target bound per gram of the polymer, A is the prefactor, *C_f_* is the free concentration (mM) and ν reflects the heterogeneity of the system, indicating how much it would deviate from a standard linear fit where ν equals 1.

Equation (1) corresponds to the Freundlich isotherm and is often used for fitting of MIP binding isotherms as the distribution of the binding sites and affinity constants are assumed to be heterogeneous [32,33]. At *C_f_* = 0.3 mM, the IF was 3.1 ± 0.1 while higher concentrations gave slightly lower IF values (~2.5) due to saturation of the binding sites. The obtained results were comparable to other dopamine MIPs in literature [25,26]. Such response of the MIP towards the competitor serotonin was not significantly different than the reference, demonstrating the selectivity of the system.

### 2.2. Cyclic Voltammetry Measurements

Through optical batch rebinding experiments, the specificity and selectivity of the MIP towards its template dopamine were demonstrated. Subsequently, the MIP particles were functionalized into SPEs by incorporating them into the bulk screen-printing ink at different ratios (0%–30%; see experimental section). An SEM image of a MIP-SPE is provided in Figure S2. The electrochemical oxidation of dopamine has been extensively researched and is a key analytical probe for the benchmarking of innovative designs and modifications upon carbon surfaces [31]. Following the modifications of the SPEs, with a maximum ratio of particles *vs.* ink, cyclic voltammetry measurements were performed and compared to the results of bare/unmodified SPEs.

**Figure 2:**
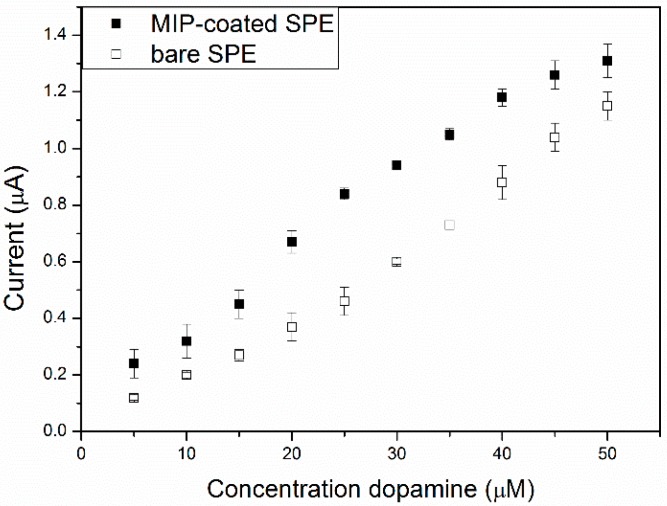
The effect of increasing dopamine concentrations (0–50 μM) made into a pH 7.4 PBS using both bare SPE (open squares) and MIP-SPE (filled squares) The average response is shown and the corresponding standard deviation (*N* = 3) are presented in the error bars.

In Figure S3, the cyclic voltammograms of the bare SPEs and a MIP-SPE are shown when the electrodes were exposed to PBS solutions of increasing dopamine concentration (0–50 μM, with steps of 5 μM). A clear response was observed in the dopamine oxidation peak, located at +0.2 V. For the MIP-SPEs a minor shift (+0.17 V) occurred, which was likely due to the increase in surface area caused by the particles. Analysis of the oxidation peak height *vs.* dopamine concentration showed that the response in both electrodes was linear and a calibration curve was constructed (Figure 2).

The response of both electrodes towards the detection of dopamine is linear over the concentration range of 0–50 µM (*R*^2^ = 0.97), indicating the sensitive regime utilizing such sensor platform. For the bare SPEs, the gradient was 0.023 μA/μM dopamine, while for the MIP-SPE this was equal to 0.025 μA/μM dopamine. The limit of detection, defined as the concentration at which the signal is three times the standard deviation, was then determined. This corresponded to 4.7 ± 0.05 μM for the MIP-SPE and respectively 4.0 ± 0.06 μM for the bare SPE, which is in the competitive range of graphitic screen-printed platforms reported in literature [34]. MIP particles are non-conductive and therefore reduce the number of conductive pathways, hence likely explaining the adverse effect on detection with cyclic voltammetry. However, we will show that this functionalization procedure will significantly enhance thermal detection.

### 2.3. Heat-Transfer Method (HTM) Measurements on Dopamine in Buffer Solutions with MIP-SPE

HTM measurements were performed on SPEs modified with different ratios of MIP particles *vs.* ink, respectively 5% and 30%. For comparison, the results with 5% and 30% are provided in Figure S4. It shows that there was a significant difference in performance and 30% was determined to be the best configuration. All further experiments will therefore be conducted with these electrodes.

**Figure 3:**
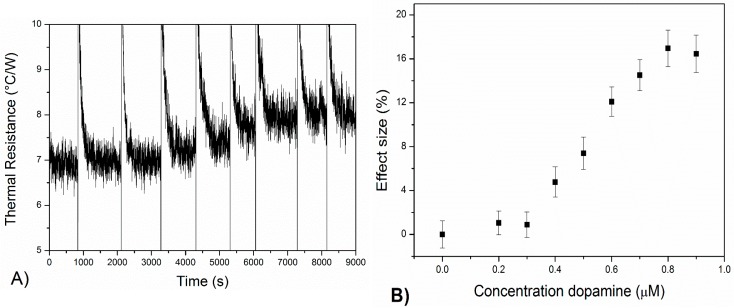
Time-dependent thermal resistance data (panel **A**). At constant *T_1_*, the temperature inside the liquid of the flow cell (*T_2_*) clearly decreased with solutions of increasing dopamine concentrations (pH = 7.4) due to blocking of heat-transfer in the MIP. The linear regime of the sensor lies from 0.3 μM to 0.8 μM (**B**), and the sensor has a maximum effect size of 16.5% ± 1%. Error bars were calculated as the standard deviation over a 500 s window after each addition, measurements performed in threefold.

To measure the MIP-SPE with HTM, the set up was placed in an environment with a stable ambient temperature of 20 ± 0.02 °C. The temperature of the copper block, *T_1_*, was strictly controlled at 37 ± 0.02 °C by a PID controller. First, the flow cell was filled with pure PBS buffer solution (pH = 7.4) and after stabilization of T_2_, PBS solution with increasing concentration of dopamine were added (0–1000 nM). When these concentrations were added this resulted in a quick drop within *T_2_*, which was rapidly stabilized by the PID controller. After reaching a stable plateau level, the sensor cell was left to stabilize for at least 15 min after each addition. The decrease in *T_2_* can then solely be attributed to the binding of the target molecules to the MIP layer. In Figure 3A, the time-dependent thermal resistance values are shown and Figure 3B shows the corresponding *R_th_* data with its dose-response curve. The normalized values were calculated by dividing the *R_th_* after each addition to the base-line signal.

Figure 3 demonstrates that the thermal resistance increased stepwise from 6.80 ± 0.10 °C/W to 7.92 ± 0.09 °C/W by gradually increasing the dopamine concentration to 900 nM within PBS. This corresponded to a percentage increase of 16.5%, significantly higher than that of the noise on the signal (1.1%) and thereby proving that the effect was caused by binding of the target to the nanocavities. In addition, it is proven that the MIP is specific towards dopamine since for the reference NIP (Figure S4) the thermal resistance did not significantly change with increasing concentrations of the template.

The obtained dose–response curve in Figure 3B showed typical behavior; at concentrations until 800 nM the binding effect increased linearly with the concentration while at higher concentrations a trend towards saturation was exhibited which can be attributed to increasing occupation of the binding sites. With the linear fit, the limit of detection was determined to be 350 ± 30 nM which is a significant improvement compared to cyclic voltammetry (4700 ± 50 nM). On the same sample, simultaneously with HTM, measurements with the novel concept TWTA were performed.

### 2.4. Thermal Wave Transport Analysis (TWTA) Measurements on Dopamine in Buffer Solutions with MIP-SPE

**Figure 4:**
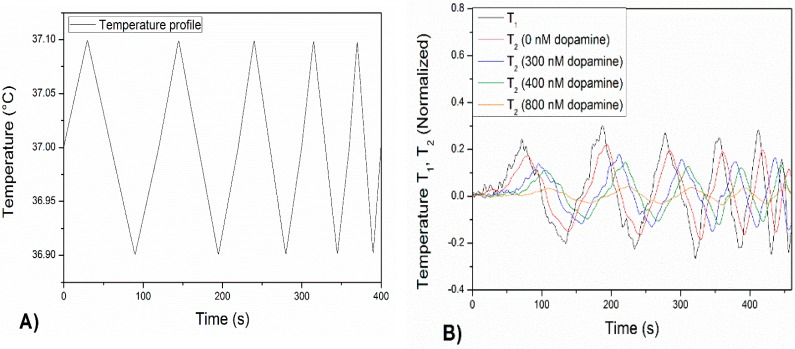
A thermal wave was applied by the heat sink and the MIP-SPE (with ratio M_P_/M_I_ 30%) were exposed to a dopamine-free PBS solution and subsequently to solutions with dopamine concentrations of 300, 400 and 800 nM in PBS (pH = 7.4). The wave has an amplitude of 0.1 °C and an increasing frequency, ranging from 0.01 to 0.05 Hz (**A**). The resulting heat wave (*T_2_*) inside the liquid compartment was followed in time with increasing concentrations of dopamine in PBS and normalized to the initial temperature of 37.00 °C (**B**).

Besides analyzing the heat-transport through the functionalized chip, the phase shift in response to the heat sink was studied simultaneously. To this extent, on the same sample as on which the heat-transfer was applied, at certain points in time thermal waves were applied by the heat sink. First, the chip was exposed to a dopamine-free PBS solution and subsequently to increasing dopamine concentrations of 300, 400 and 800 nM in PBS. The applied thermal waves had an amplitude of 0.1 °C and an increasing frequency, ranging from 0.01 to 0.05 Hz (Figure 4A). It has to be noted that due to the minor amplitude of the thermal wave, it was ensured that this did not interfere with HTM measurements. The resulting heat wave (*T_2_*) inside the liquid compartment was monitored in time at increasing concentrations of dopamine and normalized to the initial temperature of 37.00 °C (Figure 4B).

**Figure 5:**
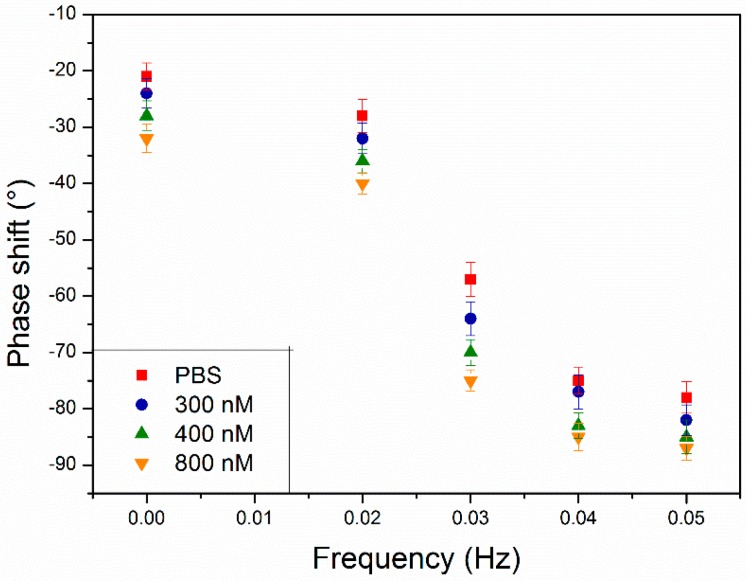
The phase shifts (Hz) for the concentrations of dopamine (0, 300, 400, 800 nM) in PBS solutions (pH = 7.4) as a function of the frequency. The highest response was achieved at 0.03 Hz, demonstrating this would be the optimum frequency to monitor dopamine binding to the MIP particles.

In Figure 4B, the black line corresponds to the input temperature wave measured by the thermocouple located inside the heat sink (*T*_1_). The red line represents the resulting heat wave in the liquid (*T*_2_) when the MIP-SPE were exposed to a pure PBS buffer solution. The phase shift observed between the black and red curve was due to the time required to transfer heat from the heat sink to the center of the liquid compartment. A slight increase of the phase shift, accompanied with a decrease of the amplitude of the signal, was observed when a 300 nM solution of dopamine (blue line) within a PBS was added to the set up. With higher concentrations of dopamine, the effect became more pronounced and the measured phase shift increased. From the HTM measurements was concluded that binding of the neurotransmitter to the MIP-layer resulted in a rise in the heat-transfer resistance at the solid-liquid interface. This leads to slower dissipation of the heat from the heat sink to the liquid compartment and explains the results observed in Figure 4A. The observed phase shifts (Figure 4B) were quantified and summed up in Figure 5. For each frequency, the error bars were calculated by subtracting the measured results with a fitted curve.

From Figure 5 it can be concluded that 0.03 Hz is the optimal frequency to measure the target-receptor dynamics and should be used within future experiments. From 300 nM onwards, a significant effect in the thermal wave output was measured. At this optimal frequency, a phase shift of −57° ± 1° was observed in PBS, while at 800 nM this increased to −75° ± 2°, which corresponded to a percent increase of 31% ± 2%. Simultaneously with analyzing the thermal wave output, HTM measurements were performed on the same substrate. However, with HTM a higher detection limit (350 nM) was determined and at the same concentration of 800 nM, the effect size was only 16% ± 1%, which is nearly a factor of two lower than for TWTA. This demonstrated the benefits of the new technique and showed that by optimizing the signal-to-noise ratio, neurotransmitter detection was enhanced. Specificity of the sensor platform was demonstrated by measurements conducted on the NIP, where no significant difference in phase shift was observed with solutions of increasing concentrations (Figure S5).

### 2.5. Thermal Wave Transport Analysis (TWTA) Measurements on Dopamine in Food Sample with MIP-SPE

**Figure 6:**
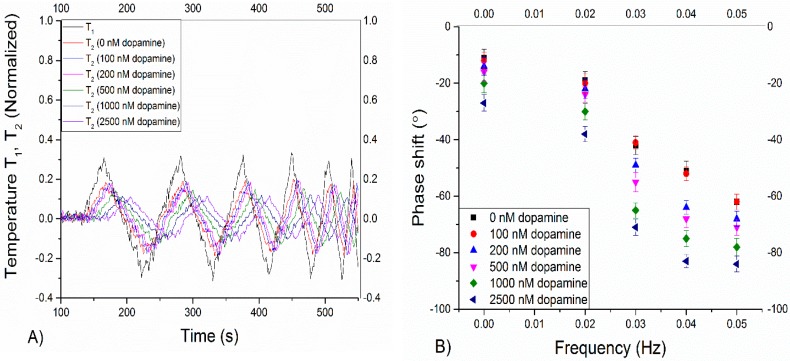
A thermal wave (amplitude 0.1 °C) was applied by the heat sink and the MIP-SPE were exposed to a dopamine-free solution of banana and subsequently to banana fluid with spiked dopamine concentrations of 100, 200, 500, 1000 and 2500 nM. The results on the thermal wave output normalized to the initial temperature of 37.00 °C are shown in (**A**), while (**B**) provides the corresponding phase shifts. 0.03 Hz was determined to be the optimal frequency for the measurements, as was observed in previous experiments.

Certain foods, such as bananas, contain a high content of dopamine (up to 8 mg/kg banana) and therefore measurements of food samples is extremely relevant [30]. For the HTM measurements, bananas were ground, centrifuged and filtered in order to obtain a clear liquid. The resulting solution was then spiked with solutions of increasing dopamine content (0 nM to 2500 nM) and HTM and TWTA measurements were performed with both the MIP and its reference NIP (Figure S6). The results of only the HTM measurements are shown in Figure S7, showing that only from spiked concentrations of 500 nM and higher a significant effect on the thermal resistance was performed. For the TWTA measurements, the result of the thermal wave outputs normalized to the initial temperature of 37.00 °C and corresponding phase shifts are given in Figure 6. A gentle filter (10 point median) was applied to the data. This has been done previously with other biological samples in order to correct for viscosity effects [10]. At each frequency, the error bars were calculated by subtracting the measured results from a fitted curve.

It is not an easy task to measure food related samples because of their high viscosity and the presence of other interfering compounds, such as large proteins. In previous experiments, it has been demonstrated that this had a significant effect upon the limit of detection due to the increase of non-specific binding and higher noise levels for HTM [10]. On the sample as shown in Figure 6 HTM was performed, and this resulted in measurable effects from spiked concentrations of 500 nM and higher. In the case of TWTA, effect size is enhanced and a significant phase shift was observed between results of a pure non-spiked solution (−41 ± 1 Hz) and a solution spiked with 200 nM of dopamine (−49 ± 2 Hz).

The maximal percentage increase is observed at 2500 nM where the phase decreases to −83 ± 2 Hz whereas in the buffer this is around 60 Hz, corresponding to an effect size of roughly 38%. This is a combination of the effect of the spiked dopamine concentration and of the initial dopamine present in the banana. If this is compared to the response to the reference NIP (Figure S6), there no significant difference in the phase behavior is observed which demonstrates the specificity of the sensor platform. Since the spiked concentration of 200 nM that can be detected is similar to concentrations present in food samples [27,28,29], this provided proof-of-application of the developed bio-sensing platform with TWTA as transduction technique.

## 3. Methods

### 3.1. Materials

Ethylene glycol dimethacrylate (EGDMA), methacrylic acid (MAA), dopamine hydrochloride salt (99%), and methanol were purchased from Acros (Loughborough, Leicestershire, UK). Prior to polymerization, the stabilizers in the MAA and EGDMA were removed by passing the solutions over a column packed with alumina. 4,4’-Azobis(4-cyanovaleric acid) and serotonin creatinine sulfate monohydrate (98%) were purchased from Sigma Aldrich (Gillingham, UK). For the heat-transfer measurements, a 1× phosphate buffered saline (PBS) solution was prepared with Dulbecco tablets obtained from Oxoid Limited (Basingstoke, UK).

### 3.2. Synthesis of a MIP for Dopamine

For the synthesis of the dopamine MIP, the procedure described by Lulínski *et al.*, was adapted [25]. The MIP was prepared as follows: first, a mixture of MAA (0.54 g, 6.6 mmol), EGDMA (2.96 g, 14.9 mmol), and 4,4’-azobis(4-cyanovaleric acid) (65 mg) was dissolved in methanol (3.67 mL) and water (0.57 mL) together with the template molecule dopamine (0.063 g, 0.33 mmol). This solution was degassed with N_2_ and heated up to initiate polymerization. To allow full completion of the reaction, the mixture was kept at 65 °C for 12 h. After polymerization, the bulk polymer was ground and sieved to obtain microparticles with sizes smaller than 10 µm. Finally, the dopamine was removed from the MIP powders by continuous extraction with a 50/50 mixture of methanol and water. After 6 h, the template was fully extracted which was verified by AT-IR spectroscopy with a Nicolet 380 FT-IR device from Thermo Scientific (Loughborough, Leicestershire, UK). (Figure S1 in Supplementary Information). Subsequently, the powders were dried in an oven for 12 h at 100 °C. A non-imprinted polymer (NIP) was synthesized accordingly, but without the presence of the target molecule. These MIP and NIP powders were used in all further batch-rebinding and heat-transfer experiments.

### 3.3. Batch Dopamine Binding Experiments: Analysis by UV-vis

The specificity and binding isotherms of the MIP and NIP particles were determined by optical batch binding experiments with an Agilent 8453 spectrophotometer (Stockport, UK). For the experiments, MIP or NIP powder (20 mg) was added to aqueous dopamine solutions (5 mL) in the concentration range between 0.3 to 1.0 mM. The resulting suspensions were shaken for 12 h on a rocking table at room temperature. Subsequently, the suspensions were filtered and the free concentration of dopamine (*C_f_*) was determined by UV-vis spectroscopy. From this, the bound concentration (*S_b_*) of dopamine was calculated per gram of MIP and NIP and binding isotherms were obtained. By fitting the binding isotherms, the specificity of the MIP towards the template dopamine was determined. To test the selectivity, the competitor molecules serotonin was used since its structure is very similar to dopamine. For these experiments, 20 mg of MIP powder was added to 5 mL of aqueous serotonin solutions and binding isotherms were determined after filtration of the suspensions.

### 3.4. Preparation of MIP Bulk Modified Screen-Printed Electrodes (MIP-SPEs)

Experiments carried out throughout this study utilize screen-printed electrodes (SPEs) (41 mm × 7 mm) which comprise of a three-electrode configuration with a 3 mm graphite working electrode, a graphite counter electrode and an Ag/AgCl pseudo-reference electrode. These SPEs were fabricated in-house with appropriate stencil designs to achieve a 3 mm diameter working electrode respectively, using a microDEK 1760RS screen-printing machine (DEK, Weymouth, UK). Firstly, a carbon-graphite ink formulation (Product Code: C2000802P2; Gwent Electronic Materials Ltd., Pontypool, Wales, UK) was printed onto a polyester (Autostat, Utrecht, The Netherlands, 250 micron thickness) substrate. This layer was then cured in a fan box oven with extraction at 60 °C for 30 min. Next a silver/silver chloride reference electrode was included by screen-printing Ag/AgCl paste (Product Code: C2040308P2; Gwent Electronic Materials Ltd.) onto the substrate. Finally, a dielectric paste (Product Code: D2070423D5; Gwent Electronic Materials Ltd.) was printed onto the polyester substrate to cover the connections. After curing at 60 °C for 30 min, the SPEs were ready to be used. Prior to functionalization of the SPEs with the MIPs particles, the reproducibility of this batch of sensors was found to correspond to less than 4% RSD towards the redox probe, [Ru(NH_3_)]^2+/3+^/0.1 M KCl, utilizing an edge connector [35]. The MIPs were incorporated into the bulk ink of the SPEs on the basis of the weight percent of M_P_ and M_I_, where M_P_ is the mass of particulate and M_I_ is the mass of the ink formulation used in the printing process [36,37]. Typically, the weight percent of M_P_ and M_I_ varied in the range of 0%–30% (M_P_/M_I_). At higher weight percentages, the ink loses its printability because the concentration of graphite becomes too low.

### 3.5. Cyclic Voltammetry Measurements of SPEs

Cyclic voltammetric measurements were carried out using an Autolab PG-STAT potentiostat (Metrohm, Utrecht, The Netherlands). The experiments performed throughout this study consisted of a three electrode system. Graphitic screen-printed electrodes (standard-SPE) and MIP-SPE were used as the defined working electrodes, with the addition of a platinum counter and a saturated calomel electrode (SCE) as the reference electrode to complete the circuit. This electroanalytical protocol was studied over solutions of increasing dopamine concentrations (0–50 µM, steps of 5 μM) within a nitrogen degassed (pH = 7.4) PBS solution. The electrochemical oxidation peak at ~+0.20 V was used as the analytical parameter [31]. This experimental procedure was carried out over the potential range of −0.2 to +0.8 V at a scan rate of 50 mV·s^−1^.

### 3.6. Design of Set up for Thermal Measurements

The equipment used for the thermal resistance measurements featured an in-house design and was described previously in [10,38]. A Perspex flow cell with an inner volume of 110 µL (6 mm diameter, 4 mm and inner height) was coupled to the system which was sealed off with an O-ring with a contact area of 28 mm^2^. The MIP-SPE were mounted horizontally in the set up and pressed mechanically onto a copper block, which served as a heat sink. The temperature of the copper, *T*_1_, was actively steered by a proportional-integral-derivative (PID) controller (*P* = 8, *I* = 1, *D* = 0) and measured by a thermocouple. For HTM measurements of neurotransmitters, this temperature was kept constant at 37.00 °C. Above the sensor chip surface a second thermocouple was positioned, which measured the temperature in the liquid, *T*_2_ The thermal resistance, abbreviated as *R_th_* (°C/W), was determined by dividing the temperature difference (*T*_1_ − *T*_2_) over the input power *P* (W) that is required to keep the temperature constant at 37.00 °C (Equation (2)).

(2) $$R_{th}=\;\frac{T_{1}-T_{2}}{P}$$

**Figure 7:**
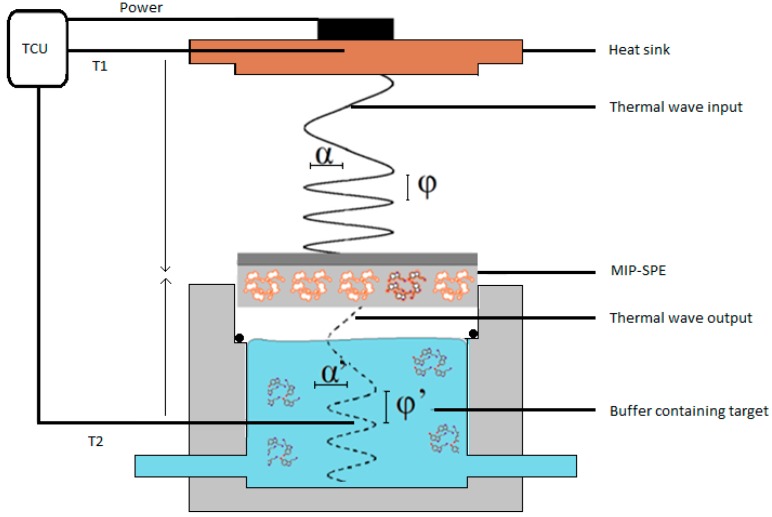
Schematic representation of the thermal wave analysis transport set up, with TCU corresponding to the thermal control unit. The thermal wave (with phase ϕ) is sent through the MIP-SPE and the output signal (ϕ′) is subsequently measured in the liquid.

The MIP-SPE were first stabilized into PBS solutions and then solutions of increasing dopamine concentrations (0–900 nM) in PBS were added into the flow cell. The flow cell used was the same as for the HTM measurements and is described in detail in [35]. After stabilization of the signal, the *R_th_* values at the specified concentrations were determined and corresponding dose-response curves were constructed. Simultaneously with these measurements, a novel thermal technique was employed, which is based on the thermal wave transport analysis (Figure 7).

A thermal wave (amplitude 0.1 °C, frequency increased from 0.01 to 0.05 Hz) was applied from the heat sink to the MIP-SPE and the output was measured in the liquid at *T*_2_. This was dependent upon the amount of target bound onto the MIP interface, which induces a delay in the phase (ϕ ≠ ϕ′) and a decrease in amplitude (α ≠ α′) of the signal.

Thermal wave transport analysis was performed simultaneously with the standard HTM measurements on the MIP-SPE. Instead of keeping *T*_1_ at a fixed temperature required to determine the heat-transfer resistance, at four chosen dopamine concentrations in PBS solutions (0, 300, 400, 800 nM) the PID controller for TWTA measurements up transmitted a thermal wave through the heat sink by means of an adjustable heat source. The corresponding wave had an amplitude of 0.1 °C and frequency was increased from 0.01 to 0.05 Hz. When dopamine was bound to the MIP particles, this induced a delay in the phase (ϕ ≠ ϕ′) and a decrease in amplitude (α ≠ α′) of the thermal wave output measured at *T*_2_. Since the thermal wave only had an amplitude of 0.1 °C and was applied at no more than four distinct points it time, it did not affect the stability of the system or the results obtained by HTM.

In order to demonstrate its proof-of-application, a food sample was measured. This was done by grinding bananas for 4 min in a blender type Avent SCF870/20 (Philips, Eindhoven, The Netherlands) and subsequently centrifuging this mixture at 3200 pm for 5 min. The supernatant was filtered and a clear liquid was obtained, which was spiked with solutions of increasing dopamine concentrations (100, 200, 500, 1000, 2500 nM).

## 4. Conclusions

MIPs for the detection of dopamine were synthesized and the resulting reaction mixtures were ground in order to obtain micron-sized particles. With optical batch rebinding experiments, the specificity and selectivity of the MIPs towards its target molecule dopamine were demonstrated. Next, these MIP particles were functionalized within a SPE with a novel and more straightforward procedure that is based on direct mixing of the particles with the screen-printing ink. The optimal ratio of particles *vs.* ink was determined to be 30%. Subsequently, the limits of detection of dopamine were determined with the optimized MIP-modified electrodes by three independent techniques. Cyclic voltammetry was used as electrochemical method and two thermal techniques were applied, respectively the heat-transfer method that measures the thermal resistance at the solid-liquid interface, and a novel strategy that is based on the analysis of thermal waves. The results, for both dopamine in buffer solutions and within a spiked food sample, are summarized in Table 1.

From Table 1, it is directly clear that thermal methods are an interesting alternative to electrochemical methods since the limit of detection in buffer solutions increased by an order of magnitude and it gives the possibility to measure complex food samples. Compared to HTM, analyzing the transport of thermal waves had a significantly higher effect size (31% *vs.* 16% at 800 nM in dopamine buffer solutions) and enhanced the detection limit by requiring less stringent temperature control.

In summary, two novel concepts were described which made improvements both on the functionalization side and on the sensing aspect. The direct mixing of MIP particles with screen-printing ink eliminated the need of extra preparative steps and ensured functionalization of electrodes in mass-production. On the sensing side, for the first time, we reported on thermal wave transport analysis (TWTA) that resulted in limits of detection for dopamine in the nanomolar regime for not only buffer solutions, but also with a relevant food sample. An additional benefit is that this technique can be performed simultaneously with the heat-transfer method, allowing direct validation of the results. With the developed MIP-based sensor platform being generic, the described methodology offers a new approach for the fast and cost-effective detection of neurotransmitters, which has interesting applications in the field of biomedical research.

## Figures and Tables

**Table 1:** Detection limits for MIP-modified SPEs of dopamine in buffer solutions and in a food sample for three different detection methods, respectively cyclic voltammetry, and the thermal methods HTM, and TWTA.

Detection Method	LOD Buffer Solutions (nM)	LOD Food Sample Spiked with Dopamine (nM)	Sample Preparation Time (min)	Sample Measurement Time (min)
Cyclic voltammetry	4700 ± 50	-	1	2
Heat-transfer method (HTM)	350 ± 30	500 ± 50 nM	45	15–20
Thermal wave transport analysis	260 ± 35	150 ± 40 nM	1	3–5

The sample preparation time is associated with the preparation time per sample. In the case of screen-printing, roughly 60 electrodes are produced within 1 h.

## Supplementary Materials

The following are available online at www.mdpi.com/1420-3049/21/5/552/s1. Supporting information to this document contains additional graphs that are referred to in the text.

## References

[1] Wulff G. (2002). Enzyme-like catalysis by molecularly imprinted polymers. Chem. Rev..

[2] Haupt K., Mosbach K. (2000). Molecularly imprinted polymers and their use in biomimetic sensors. Chem. Rev..

[3] Matsui J., Nicholls I.A., Takeuchi T., Mosbach K., Karube I. (1996). Molecularly-imprinted polymeric logic gates selective for predetermined chemical input species. Anal. Chim. Acta.

[4] Bossi A., Bonini F., Turner A.P.F., Piletsky S.A. (2007). Molecularly imprinted polymers for the recognition of proteins: The state of the art. Biosens. Bioelectron..

[5] Yano K., Karube I. (1999). Molecularly imprinted polymers for biosensor applications. TrAC Trends Anal. Chem..

[6] Ye L., Haupt K. (2004). Moleclarly imprinted polymers as antibody and receptor mimics for assays, sensors and drug discovery. Anal. Bioanal. Chem..

[7] Pietzryk A., Suriyanarayanan S., Kutner W., Maligaspe E., Zandler M.E., D’Souza F. (2010). Molecularly imprinted polymer (MIP) based piezoelectric microgravimetry chemosensor for selective determination of adenine. Biosens. Bioelectron..

[8] Maouche N., Ktari N., Bakas I., Fourati N., Zerrouki C., Seydou M., Maurel F., Chehimi M.M. (2015). A surface acoustic wave sensor functionalized with a polypyrrole molecularly imprinted polymer for selective dopamine recognition. J. Mol. Recognit..

[9] Matsui J., Akamatsu K., Hara N., Miyoshi D., Nawafune H., Tamaki K., Sugimoto N. (2005). SPR sensor chip for detection of small molecules using molecularly imprinted polymer with embedded gold nanoparticles. Anal. Chem..

[10] Peeters M., Csipai P., Geerets B., Weustenraed A., van Grinsven B., Gruber J., de Ceuninck W., Cleij T.J., Troost F.J., Wagner P. (2013). Heat-transfer-based detection of l-nicotine, histamine, and serotonin using molecularly imprinted polymers as biomimetic receptors. Anal. Bioanal. Chem..

[11] Van Grinsven B., Vanden Bon N., Strauven H., Grieten L., Murib M., Jiménez-Monroy K.L., Janssens S.D., Haenen K., Schöning M.J., Vermeeren V. (2012). Heat-transfer resistance at solid-liquid interfaces: A tool for the detection of single-nucleotide polymorphisms in DNA. ACS Nano.

[12] Van Grinsven B., Eersels K., Peeters M., Losada-Pérez P., Vandenryt T., Cleij T.J., Wagner P. (2014). The heat-transfer method: A versatile low-cost, label-free, fast, and user-friendly readout platform for biosensor applications. ACS Appl. Mater. Interfaces.

[13] Bers K., Eersels K., van Grinsven B., Daemen M., Bogie J., Hendriks J., Bouwmans E., Püttmann C., Stein C., Barth S. (2014). Heat-transfer resistance measurement method (HTM)-based cell detection at trace levels using progressive enrichment approach with highly selective cell-binding surface imprints. Langmuir.

[14] Peeters M., Kobben S., Jiménez-Monroy K.L., Modesto L., Kraus M., Vandenryt T., Gaulke A., van Grinsven B., Ingebrandt S., Junkers T. (2014). Thermal detection of histamine with a graphene oxide based molecularly imprinted polymer platform prepared by reversible addition-fragmentation chain transfer polymerization. Sens. Actuator B Chem..

[15] Thoelen R., Vansweevelt R., Duchateau J., Horemans F., D’Haen J., Lutsen L., Vanderzande D., Ameloot M., vandeVen M., Cleij T.J. (2008). A MIP-based impedimetric sensor for the detection of low-MW molecules. Biosens. Bioelectron..

[16] Honeychurch K.C., Hart J.P. (2003). Screen-printed electrochemical sensors for monitoring metal pollutants. TrAC Trends Anal. Chem..

[17] Wilson R., Turner A.P.F. (1992). Glucose oxidase: An ideal enzyme. Biosens. Bioelectron..

[18] Metters J.P., Kadara R.O., Banks C.E. (2011). New directions in screen printed electroanalytical sensors: An overview of recent developments. Analyst.

[19] Andreescu S., Barthelmebs L., Marty J.-L. (2002). Immobilization of acetylcholinesterase on screen-printed electrodes: Comparative study between three immobilization methods and applications to the detection of organophosphorus insecticides. Anal. Chim. Acta.

[20] Croux D., Vangerven T., Broeders J., Boutsen J., Peeters M., Duchateau S., Cleij T.J., Deferme W., Wagner P., Thoelen R. (2013). Molecular imprinted polymer films on RFID tags: A first step towards disposable packaging sensors. Phys. Status Solidi A.

[21] Kröger S., Turner A.P.F., Mosbach K., Haupt K. (1999). Imprinted Polymer-Based Sensor System for Herbicides Using Differential-Pulse Voltammetry on Screen-Printed Electrodes. Anal. Chem..

[22] Pellicer C., Gomez-Caballero A., Unceta N., Aranzazu Goicolea M., Barrio R.J. (2010). Using a portable device based on a screen-printed sensor modified with a molecularly imprinted polymer for the determination of the insecticide fenitrothion in forest samples. Anal. Methods.

[23] Zang D., Yan M., Ge S., Ge L., Yu J. (2013). A disposable simultaneous electrochemical sensor array based on a molecularly imprinted film at a NH_2_-graphene modified screen-printed electrode for determination of psychotropic drugs. Analyst.

[24] Kirsch N., Hart J.P., Bird D.J., Luxton R.W., McCalley D.V. (2001). Towards the development of molecularly imprinted polymer based screen-printed sensors for metabolites of PAHs. Analyst.

[25] Lulínski P., Maciejewska D., Bamburowicz-Klimkowska M., Szutowski M. (2007). Dopamine-imprinted polymers: Template-monomer interactions, analysis of template removal and application to solid phase extraction. Molecules.

[26] Suedee R., Seechamnanturakit V., Suksuwan A., Canyuk B. (2008). Recognition properties and competitive asays of dual dopamine/serotonin selective molecularly imprinted polymer. Int. J. Mol. Sci..

[27] Fienberg A.A., Hiroi N., Mermelstein P.G., Song W.-J., Snyder G.L., Nishi A., Cheramy A., O’Callaghan J.P., Miller D.B., Cole D.G. (1998). DARPP-32: Regulator of the efficacy of dopaminergic neurotransmission. Science.

[28] Davis K.L., Kahn R.S., Ko G., Davidson M. (1991). Dopamine in schizophrenia: A review and reconceptualization. Am. J. Psychiatry.

[29] Javitt D.C., Zukin S.R. (1991). Recent advances in the phencyclidine model of schizophrenia. Am. J. Psychiatry.

[30] Wuyts N., Lognay G., Verscheure M., Marlier M., de Waele D., Swennen R. (2007). Potential physical and chemical barriers to infection by the burrowing nematode *Radopholus similis* in roots of susceptible and resistant banana (Musa spp.). Plant Pathol..

[31] Zhang M., Liao C., Yao Y., Liu Z., Gong F., Yan F. (2014). High-performance dopamine sensors based on whole graphene solution-gated transistors. Adv. Funct. Mater..

[32] Umpleby R.J., Baxter S.C., Rampey A.M., Rushton G.T., Chen Y.Z., Shimizu K.D. (2004). Characterization of the heterogeneous binding site affinity distributions in molecularly imprinted polymers. J. Chromatogr. B Biomed. Sci. Appl..

[33] Spivak D.A. (2005). Optimization, evaluation, and characterization of molecularly imprinted polymers. Adv. Drug Deliv. Rev..

[34] Randviir E.P., Brownson D.A.C., Metters J.P., Kadara R.O., Banks C.E. (2014). The fabrication, characterization and electrochemical investigation of screen-printed graphene electrodes. Phys. Chem. Chem. Phys..

[35] Galdino F.E., Foster C.W., Bonacin J.A., Banks C.E. (2015). Exploring the electrical wiring of screen-printed configurations utilised in electroanalysis. Anal. Methods.

[36] Kadara R.O., Jenkinson N., Banks C.E. (2009). Screen printed recessed microelectrode arrays. Electroanalysis.

[37] Khairy M., Kadara R.O., Kampouris D.K., Banks C.E. (2012). Electroanalytical sensing of nitrite at shallow recessed screen printed microelectrode arrays. Electroanalysis.

[38] Van Grinsven B., Vandenryt T., Duchateau S., Gaulke A., Grieten L., Thoelen R., Ingebrandt S., de Ceuninck W., Wagner P. (2010). Customized impedance spectroscopy device as possible sensor platform for biosensor applications. Phys. Status Solidi A.

