# Supplementary material for: Introducing Thermal Wave Transport Analysis (TWTA): A Thermal Technique for Dopamine Detection by Screen-Printed Electrodes Functionalized with Molecularly Imprinted Polymer (MIP) Particles
Source: Molecules. 2016 Apr 26;21(5):552. doi: 10.3390/molecules21050552 (PMC6272947; doi:10.3390/molecules21050552)
Supplement: Supplementary file 1 [file molecules-21-00552-s001.pdf]

Molecules **2016**, *21*, 552; doi:10.3390/molecules21050552

Supplementary Materials: Introducing Thermal Wave Transport Analysis (TWTA): A Thermal Technique for Dopamine Detection by Screen-Printed Electrodes Functionalized with Molecularly Imprinted Polymer (MIP) Particles

Marloes M. Peeters, Bart van Grinsven, Christopher W. Foster, Thomas J. Cleij and Craig E. Banks

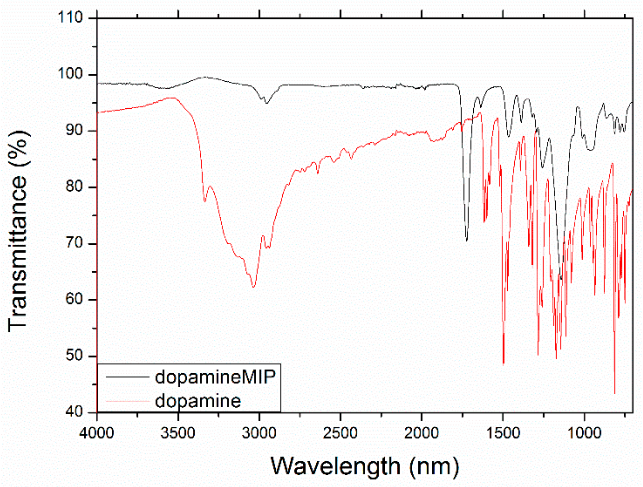

Figure S1. IR spectra of dopamine (red line) and the MIP developed for dopamine after extraction of the template (black line). The MIP showed the distinct polymer peaks at 3000 cm^{-1}, corresponding to the C-C bonds, and at 1800 cm^{-1}, which is because of the C=O functionality. The distinct peaks of the dopamine in the range of 3500–3300 cm^{-1} have disappeared, ensuring full extraction of the template molecule.

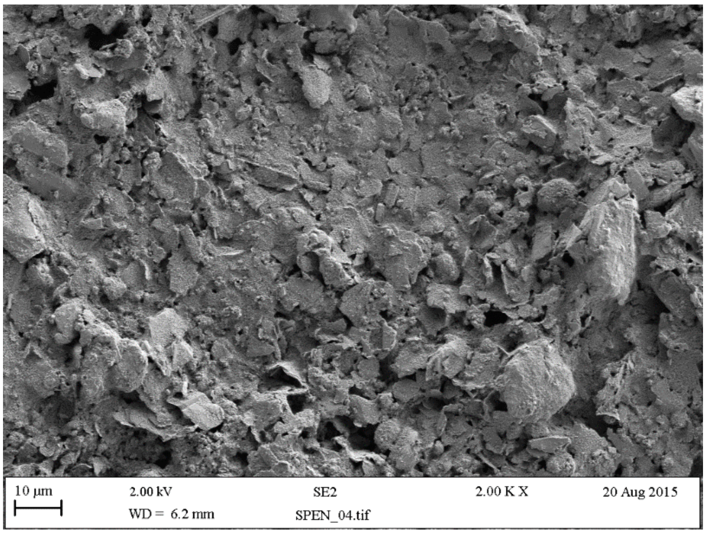

Figure S2. SEM image of SPE electrodes where the MIP particles where directly mixed in with the ink (30% MIP particles).

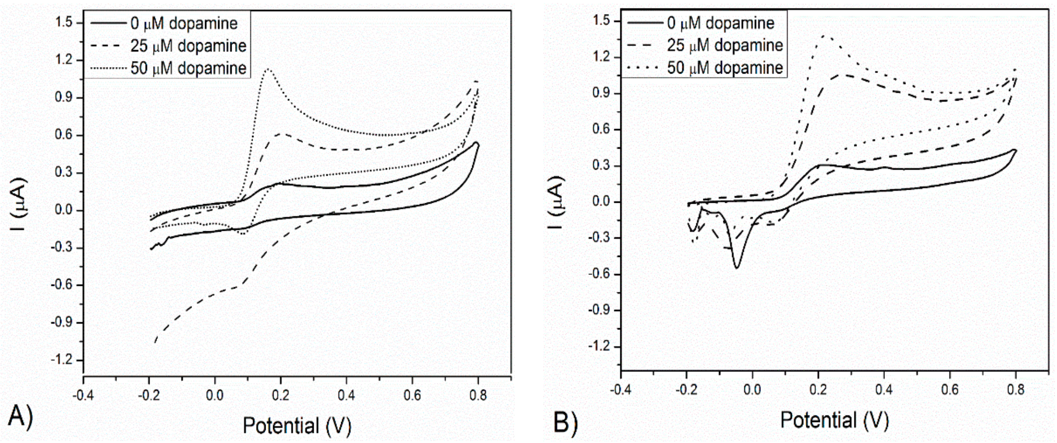

Figure S3. The response of a MIP-coated SPE (**A**) and bare SPE (**B**) to increasing concentrations of dopamine (0, 25, 50 μM) in PBS solution. The oxidation peak for the MIP-coated SPE is shifted to +0.17 V instead of +0.2 V, indicating an increase of the surface area caused by the MIPs.

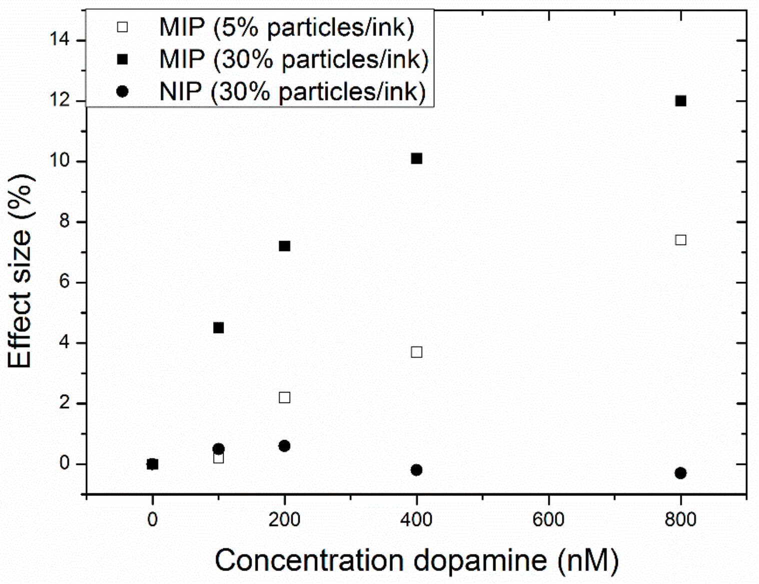

Figure S4. Shows the response (%) to concentrations of dopamine in PBS solution for the MIP mixed with 5% particles vs ink (open squares), MIP mixed with 30% particles vs ink (filled squares), and the reference NIP mixed with 30% particles vs ink (filled circles). It is clear that the response of the MIP with a 30% particles/ink ratio is significantly higher than the 5% ratio, indicating this is the optimal configuration for further experiments. In addition, since the NIP showed no significant response to the dopamine concentrations, this demonstrates the specificity of the sensor platform.

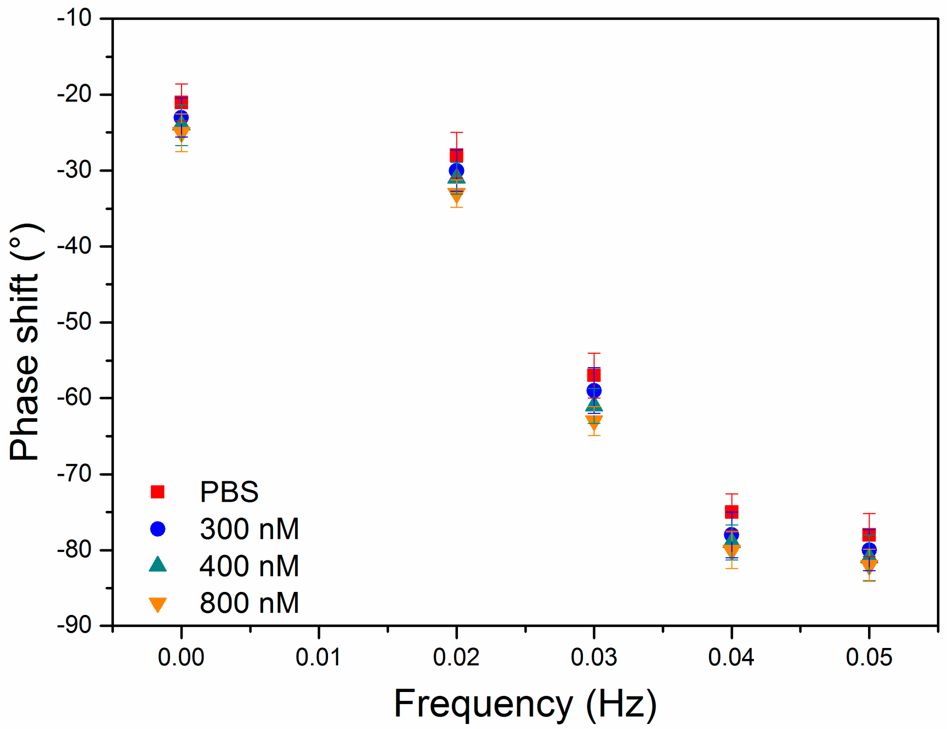

Figure S5. This shows the response of the reference NIP to buffer solutions with increasing concentrations of dopamine (0–800 nM). It is be observed that there is no significant difference in the phase shift between the thermal wave output at different dopamine concentrations.

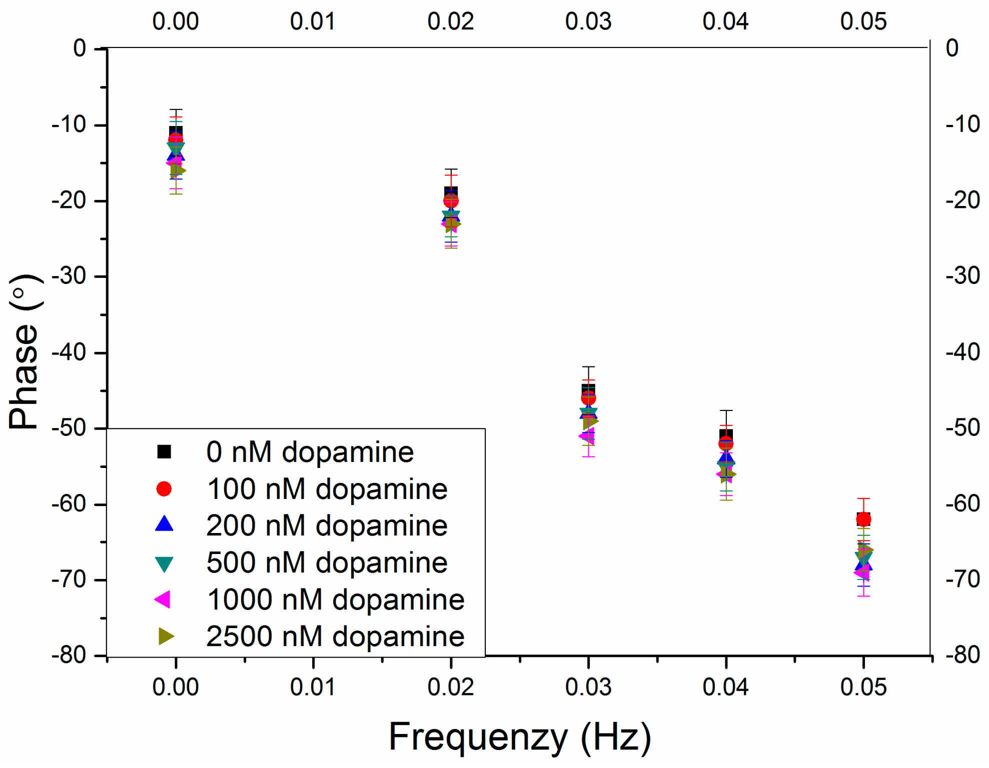

Figure S6. This shows the response of the reference NIP to banana samples that were spiked with respectively 0, 100, 200, 500, 1000 and 2500 nM of dopamine. It is observed that there is no significant difference in the phase between the thermal wave output at different dopamine concentrations.

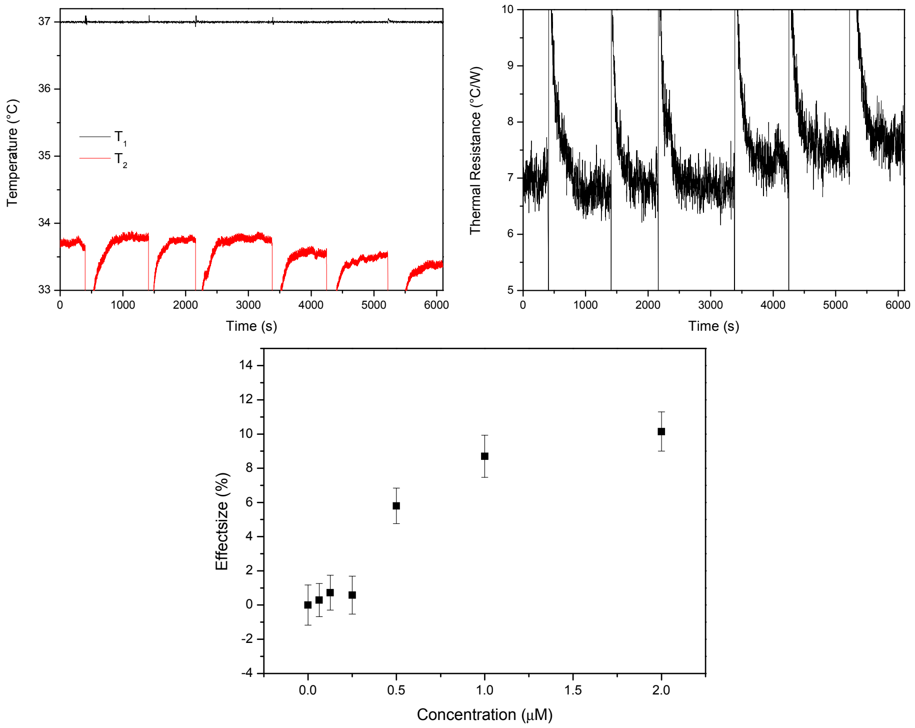

Figure S7. The top panel left shows the effect on the temperatures T_1 and T_2 upon addition of spiked solutions of dopamine (62.5 to 2000 nM) in banana fluid. The top panel right gives the raw data of the thermal resistance in time while the panel below is the determined dose-response curve. It is clear that from 500 nM, a significant effect was observed. Error bars were calculated by taking the deviation after addition on a 500 point window.
